# Modifiable risk factors remain significant causes of medium term mortality after first time Coronary artery bypass grafting

**DOI:** 10.1186/1749-8090-2-51

**Published:** 2007-12-03

**Authors:** Babu Kunadian, Joel Dunning, Russell WJ Millner

**Affiliations:** 1 Department of Cardiothoracic Surgery, The James Cook University Hospital, UK; 2 Department of Cardiothoracic Surgery, Blackpool Victoria Hospital, UK

## Abstract

**Background:**

Whilst there is much current data on early outcomes after Coronary artery bypass grafting(CABG), there is relatively little data on medium term outcomes in the current era. The purpose of this study is to present a single surgeon series comprising of all first time CABG patients operated on with the technique of cross clamp fibrillation from Feb-1996 to through to Jan-2003, and to seek risk factors for medium term mortality in these patients.

**Methods:**

Data was collected from Hospital Episode Statistics and departmental patient administration and tracking systems and cross checked using database techniques. Patient outcomes were searched using the National Health Service strategic tracing service.

**Results:**

Mean follow up was 5.3 years(0–9.4 years) and was complete for all patients. 30-day survival was 98.4%, 1-year survival 95% and 8-year survival 79%. Cox-regression analysis revealed that several modifiable pre-operative risk factors remain significant predictors of medium term mortality, including Diabetes(Hazard Ratio(HR) 1.73, 95%CI 1.21–2.45), Chromic obstructive pulmonary disease(HR 2.02, 95%CI 1.09–3.72), Peripheral vascular disease(HR 1.68, 95%CI 1.13–2.5), Body mass index>30(HR 1.54, 95%CI 1.08–2.20) and current smoker at operation(HR 1.67, 95%CI 1.03–2.72). However hypertension(HR 1.31, 95%CI 0.95–1.82) and Hypercholestrolaemia(HR 0.81, 95%CI 0.58–1.13) were not predictive which may reflect adequate post-operative control.

**Conclusion:**

Coronary artery bypass surgery using cross clamp fibrillation is associated with a very low operative mortality. Medium term survival is also good but risk factors such as smoking at operation, Chronic obstructive pulmonary disease, obesity and diabetes negatively impact this survival and should be aggressively treated in the years post-surgery.

## Introduction

The ultimate aim of coronary artery bypass grafting is to achieve long term patency of coronary bypass grafts and therefore long term symptom free survival. Therefore every technique of bypass grafting should be judged by these outcome measures. Many studies have been performed to assess the long term survival in patients undergoing coronary surgery using cardioplegic on-pump surgery[1-4] and also off-pump bypass grafting [5-7]. Indeed a recent statement from the American Heart Association compared the long term outcomes between the two techniques[8]. However there have been no studies reporting the long-term survival of patients undergoing coronary arterial bypass grafting using cross clamp fibrillation. Benefits of this technique include very short cross-clamp and bypass times, whilst consistently achieving complete revascularisation. This technique is used by 15% of UK surgeons [9-11]which equates to around 3500 operations in the UK per year. Thus we sought to assess the medium to long term survival of patients undergoing bypass grafting using cross-clamp fibrillation. Our second aim was to identify risk factors for long term mortality for these patients. Our hypothesis was that we may be able to identify risk factors pre-operatively that may be amenable to modification in the years post surgery, thereby further improving long term survival.

## Methods

### Surgical techniques

This has been previously described[11]. The heart is approached through a median sternotomy and the left Internal Mammary Artery harvested on a broad pedicle. Papaverine is topically applied. The saphenous vein is routinely used for subsequent grafts unless they are not suitable for harvesting in which case a radial artery or second mammary artery would be used. The patient is placed on cardiopulmonary bypass and the aorta cross-clamped while each distal anastomosis is fashioned. The cross clamp is then removed while the proximal anastomosis is formed. Bypass is performed at 32 degrees centigrade, and pulmonary artery venting is not routinely used.

### Data Management

Local Ethics Board Approval was obtained to utilise our own database for this study and also to obtain survival statistics for each of the patients on our database. All data was prospectively collected on the departmental Patient Access and Tracking System (PATS) (Dendrite). Survival data is available for all residents of England through the National Health Service Strategic Tracing Service, using the patients NHS number as a unique identifier. This was accessed using an Internet based, password secured approach.

Urgent patients were defined as any patient requiring their operation prior to hospital discharge. This included a small number of patients having their operation within 24 hours or referral. A good left ventricular ejection fraction (EF) was defined as an ejection fraction over 50%, moderate EF 30–50% and poor EF >30%.

### Statistics

Statistical analysis was performed on SPSS 13.0. Binomial categorical data was compared using the Fisher's test, and the remaining categorical variables were analysed using the Chi-Squared test. Continuous data was analysed using the unpaired T-test if normality could be established, and skewed or rank data was analysed using the Mann Whitney U test. Multivariate analysis was performed using Cox regression and all variables that demonstrated a univariate significant predictive ability for mortality to a p-value <0.2 were entered into the model. Of note parsonnet score was not entered into the multivariate analysis as it is a composite score of multiple potential univariate predictors, and may thus mask their predictive ability.

## Results

From Feb 1996 to through to Jan 2003, a total of 1177 patients were identified as having either elective or urgent first time isolated CABG. 80% (939/1177) of patients were male and mean age was 63 years (Standard Deviation(SD) 9.7 years). 73% had triple vessel disease, 22% had double vessel disease and 5% had single vessel disease. 17% had left main stem disease. Mean number of grafts was 3.24 (Range 1–6). Mean follow up was 5.3 years (range 0–9.4 years, minimum follow up of living patients was 892 days). The full demographics for this study are shown in table 1 together with their ability to predict medium term mortality.

**Table 1:** Study Demographics

	Total (N = 1177)	Survivors (N = 1018)	Non-long term Survivor (N = 159)	P-value
Male (%)	939 (80%)	823(81%)	116 (73%)	P = 0.026
female (%)	238 (20%)	195(19%)	43 (27%)	
Age Mean (SD)	63 years (9.7)	62 (9.6)	68 (9.8)	P < 0.0001
CCS I	124 (10%)	107 (11%)	17 (11%)	P = 0.01
II	335 (29%)	307 (30%)	28 (18%)	
III	429 (36%)	373 (36%)	56 (35%)	
IV	289 (25%)	231 (23%)	58 (36%)	
Previous MI	552 (47%)	457 (45%)	95 (60%)	P = 0.01
Prev Cardiological Intervention	87 (7.4%)	78 (7.7%)	9 (5.7%)	P = 0.42
Diabetes	203 (17%)	162 (16%)	41 (26%)	P = 0.003
Hypertension	573 (49%)	487 (48%)	86 (54%)	P = 0.012
Hypercholestrolemia	843 (71%)	749 (74%)	94 (59%)	P < 0.0005
Current smoker	73 (6.2%)	62 (6.1%)	11 (6.9%)	P = 0.723
COPD	36 (3.1%)	23 (2.3%)	13 (8.2%)	P < 0.0005
PVD	146 (12%)	110 (11%)	36 (23%)	P < 0.0005
Coronary Disease				
Single	56 (4.8%)	54 (5.3%)	2 (1.3%)	P = 0.860
Double	258 (22%)	234 (23%)	24 (15%)	
Triple	863 (73%)	730 (72%)	133 (84%)	
LMS disease	202 (17%)	173 (17%)	29 (18%)	P = 0.734
EF Good	777 (66%)	701 (69%)	76 (48%)	P < 0.0005
Fair	308 (26%)	253 (25%)	55 (34%)	
Poor	92 (8%)	64 (6%)	28 (18%)	
Pre-op IABP	17 (1.4%)	11 (1.1%)	6 (3.8%)	P = 0.019
No of distal anastomoses	3.24 (0.97) Range 1–6	3.24 (0.98)	3.26 (0.91)	P = 0.844
Bypass time	67 mins (25)	66 mins (24)	75 mins (32)	P = 0.002
X-Clamp time	27 mins (11)	27 mins (12)	27 mins (10)	P = 0.769
BMI>30	307 (28%)	259 (27%)	48 (31%)	P = 0.190
Trasylol	243 (21%)	210 (21%	33 (21%)	P = 1.000

Average cross clamp time was 27 minutes (SD 11.7) and mean bypass time was 67 minutes (SD 25). The short term complication rate was low (Table 2). The IABP usage was 1.4% (17/1177 patients), Inotropes were used in 14% (167/1177 patients). The re-operation rate was 3.5% (35/1177 patients) and the rate of sternal re-wiring was 0.7% (8/1177 patients). The readmission to ICU rate was 2% (23/1177 patients). The 30 day mortality was 1.6% (19 patients).

**Table 2:** Post operative complications

	Total (N = 1177)	Survivors (N = 1018)	Non-long term Survivor (N = 159)	P-value
IABP	14 (1.2%)	8 (0.8%)	6 (3.8%)	P = 0.001
Inotropes	167 (14%)	138 (14%)	29 (18%)	P = 0.001
Atrial Fibrillation	236 (20%)	202 (20%)	34 (21%)	P = 0.670
Blood given post-op	217 (19%)	164 (16%)	53 (34%)	P < 0.0005
Blood loss post-op (SD)	821 mls (566)	820 mls (552)	831 mls(650)	P = 0.829
Re-operation	35 (3%)	23 (2.3%)	12 (7.5%)	P = 0.001
Sternal resuturing	8 (0.7%)	2 (0.2%)	6 (3.8%)	P < 0.0005
Parsonnet (SD)	6.2 (6.2)	5.7 (5.9)	9.8 (7.0)	P < 0.0005
Renal Failure	51 (4.3%)	28 (2.8%)	23 (15%)	P < 0.0005
Reintubation	11 (0.9%)	6 (0.6%)	5 (3.1%)	P = 0.010
ICU readmission	23 (2%)	11 (1.1%)	12 (7.5%)	P < 0.0005

**Figure 1:**
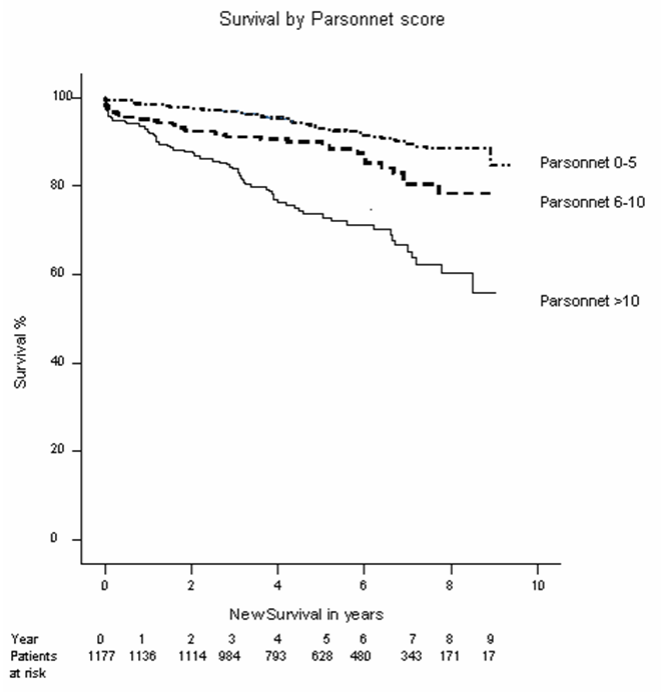
Kaplain-Meier Survival plot by Parsonnet score.

**Figure 2:**
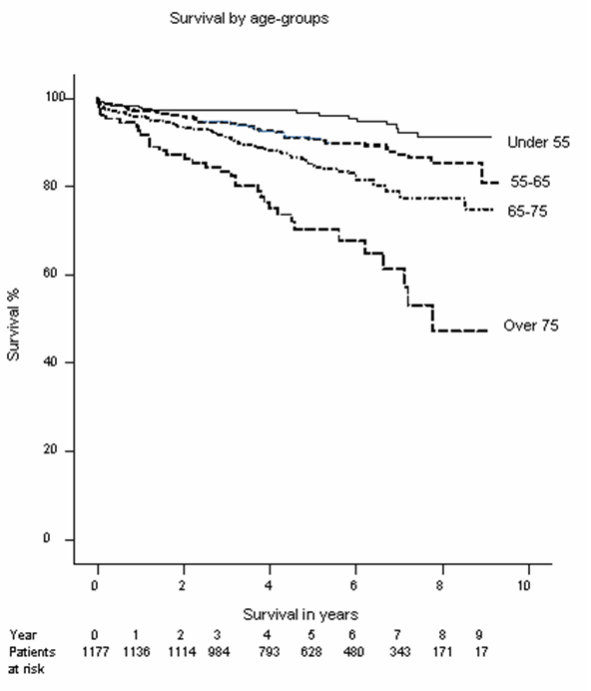
Kaplain-Meier survival plot by agegroup.

Mean follow up was 5.3 years and the 5-year survival was 86%. Follow up was performed up to 9 years and the survival at this stage was 79% across the whole group. This survival was significantly dependant on both age and parsonnet score as might be expected (Figures 1 and 2). Survival at 8 years for the various age categories were: under 55 years: 91%, 55–65 years: 83%, 65–75 years: 74%, over 75 years: 45%.

Our survival compared well with the UK survival rates of healthy residents of our region supplied by the Office of National Statistics. For example the 9 years survival of a healthy 63 year old in our region was 83%, and in our study our 9 year survival was 79% (Our mean age is 63 in our database)

We analysed the database for significant medium term predictors of mortality (Table 3). We found that several modifiable pre-operative risk factors persisted as post-operative risk factors. The presence of any type of diabetes significantly predicted adverse medium term mortality (Odds ratio(OR) 1.73, 95%CI 1.21–2.45). Also presence of chronic obstructive pulmonary disease predicted medium term mortality (Hazard Ratio(HR) 2.02, 95%CI 1.09–3.72), peripheral vascular disease predicted medium term mortality (HR 1.68, 95%CI 1.13–2.5) and a Body Mass Index(BMI) of over 30 predicted mortality(HR 1.54, 95%CI 1.08–2.20). Current smoker at operation (HR 1.67, 95%CI 1.03–2.72) also predicted medium term mortality. However hypertension (HR 1.31, 95%CI 0.95–1.82) and Hypercholestrolaemia (HR 0.81, 95%CI 0.58–1.13) failed to reach significance.

**Table 3:** Predictors of medium term mortality as determined by Cox Regression analysis

	Hazard ratio	95.0% CI for Odds ratio		Sig.
		Lower	Upper	

Diabetes	1.735	1.208	2.492	0.003
hypercholestrolaemia	0.808	0.575	1.133	0.217
Hypertension	1.317	0.953	1.821	0.096
Pre-op renal failure	5.125	2.512	10.458	<0.0005
COPD	2.017	1.094	3.718	0.025
Peripheral Vascular disease	1.677	1.127	2.495	0.011
EF 30–50%	1.483	1.026	2.143	0.036
EF <30%	2.141	1.316	3.483	0.002
BMI >30	1.540	1.079	2.198	0.017
Current smoker at operation	1.673	1.031	2.715	0.037
Age at operation	1.072	1.050	1.094	<0.0005

## Comment

We have demonstrated that coronary arterial bypass grafting using cross-clamp fibrillation is associated with low short and medium term mortality. Furthermore we have demonstrated that in the years post-cardiac surgery, modifiable risk factors remain a significant cause of mortality. Hypertension and high cholesterol did not show a significant association in our database. This may be due to the fact that these risk factors are now aggressively treated by cardiologists and general practitioners in the years post cardiac surgery. In the United Kingdom and internationally, guidelines exist for the control of both hypertension and cholesterol in the community and are regarded as being well adhered to[12,13].

In contrast diabetes, peripheral vascular disease, a high BMI, and current smoking all adversely affected long term mortality. We hypothesise that these risk factors are not as successfully treated in the years after cardiac surgery. It is our experience that patients who smoke through their admission for cardiac surgery invariably fail to stop smoking in the immediate post operative period. In addition patients with a high BMI also tend to fail to lose weight post operatively. Peripheral vascular disease may reflect patients with an aggressive atherosclerotic process in several other organs in addition to the heart and thus the risk of mortality due to other causes may be higher.

We have shown that diabetes remains a risk factor for late mortality. Further studies would be required to determine whether tighter glucose control would improve survival or whether this effect reflects the poorer prognosis of patients with diabetes although the association with tighter glucose control and survival is well established[14].

Gardner et al[15] performed a retrospective analysis of 11,815 patients undergoing coronary surgery in 43 US hospitals, searching for risk factors for 6 month survival. Their survival at 6 months was 93.8% and they found that Chronic Obstructive Pulmonary disease (COPD), cerebrovascular disease and older age predicted 6 month mortality.

Boucher et al [16] studied 329 patients over 70 years of age after cardiac surgery. They reported a 9.4% operative mortality and thereafter a 5-year survival of 86% which was comparable with the background population in their area. Their survival was comparable to that in our own study.

Caputo et al [17] reported the medium term survival of 1,479 patients who had undergone off-pump coronary arterial revascularisation. They reported a 2 year mortality of 4.7% in patients who received a complete revascularisation. Of note they found a 10.8% 2 year mortality in patients who received an incomplete revascularisation. Our 2 year survival is therefore comparable to this cohort of patients operated on with off pump surgery with complete revascularization.

The Multicenter Study of Perioperative Ischemia Research Group[18] found in an analysis of 2,048 patients from 24 centres that the 5 year survival was adversely impacted by age, diabetes, congestive heart failure and anaemia, in agreement with our own results.

Gao et al [4] analysed 56,543 patients who underwent CABG at one of 43 veterans hospitals for risk factors for long term survival. They found that diabetes had an increasing impact on survival, eventually doubling mortality at 9 years post-operatively. They also found that COPD, age and urgent surgery persisted as a risk factor in the years post surgery in agreement with our results. Their 5 year survival was 81% in their cohort of patients with the same mean age as our own.

The CASS registry followed up their cohort of 24,959 patients who underwent coronary artery bypass grafting between 1974 and 1979[2]. Their 10-year survival was 74% in this cohort of patients who had a mean age of under 55. They identified that risk factors for long term survival included heavier weight, diuretic use, diabetes and smoking. They did not investigate peripheral vascular disease as a risk factor.

Our study has weaknesses. It is retrospective in nature although our follow up is complete due to the fact that the NHS tracing service contains a complete database of all deaths in the UK. We have not yet been able to obtain data on the cause of death for our post-operative patients. In addition we have no information as to the incidence of MI, stroke or reintervention in the years post surgery. This data could potentially provide valuable additional evidence in support of the fact that modifiable risk factors may account for a large proportion of the deaths. This series is a single surgeon's series, and although this eliminates a large amount of perioperative technical heterogeneity associated with other studies, the presence of modifiable risk factors in other surgeons practice would require confirmation. Also we have not provided a detailed account of radial artery or RIMA usage although in our experience this usage was very low in this database. It is possible that long term outcomes may have been superior with arterial conduit usage although the RAPCO randomised study of saphenous vein versus arterial conduits has so far shown no benefit at 5 years after randomization [19]

We conclude that the treatment of coronary atherosclerotic disease by cardiothoracic surgeons should not end on successful discharge from the unit. The cardiothoracic surgeons should ensure that the patient is placed into programmes of smoking cessation, weight loss and diabetes supervision as appropriate. Cardiac Surgeons should ensure that these programmes are effective in their area and ensure that regular audits of their efficacy are conducted.

We have furthermore demonstrated that coronary arterial bypass grafting using cross clamp fibrillation is associated with an equivalent short and medium term survival to patients undergoing coronary revascularization either using cardioplegia or off-pump techniques.

## Abbreviations

BMI Body mass index

CABG Coronary artery bypass grafting

CI Confidence Interval

COPD Chronic Obstructive Pulmonary Disease

EF Ejection Fraction

HR Hazard ratio

IABP Intra-aortic Balloon Pump

ICU Intensive Care Unit

LMS left main stem

MI Myocardial Infarction

NHS National Health Service

OR Odds ratio

PATS Patient Access and Tracking System

PVD Peripheral Vascular disease

SD Standard Deviation

SPSS Statistical Package for Social Sciences

UK United Kingdom
